# Hypothalamic paraventricular nucleus activation contributes to neurohumoral excitation in rats with heart failure

**DOI:** 10.1186/2050-490X-2-2

**Published:** 2014-01-08

**Authors:** Yu-Ming Kang, Qing Yang, Xiao-Jing Yu, Jie Qi, Yan Zhang, Hong-Bao Li, Qing Su, Guo-Qing Zhu

**Affiliations:** Department of Physiology and Pathophysiology, Xi’an Jiaotong University School of Medicine, Xi’an Jiaotong University Cardiovascular Research Center, Xi’an, 710061 China; Department of Physiology, Nanjing Medical University, Nanjing, 210029 China

**Keywords:** Hypothalamic paraventricular nucleus, Neurohumoral excitation, Heart failure

## Abstract

Heart failure (HF) is a serious cardiovascular disease and is characterized by exaggerated sympathetic activity. In this paper, we review these limited studies, with particular emphasis on examining the role of the paraventricular nucleus (PVN) in the neurohumoral excitation in HF. The PVN is an important neuroendocrine and preautonomic output nucleus, and is considered as the important central site for integration of sympathetic nerve activity. Accumulating evidences demonstrate that a number of neurohumoral processes are involved in the pathophysiology of HF, such as renin-angiotensin system (RAS), proinflammatory cytokines (PICs), neurotransmitters, and reactive oxygen species (ROS). Recent studies about neurohumoral regulation indicate that angiotensin II type1 receptor (AT_1_-R) is the important product mediated by cytoplasmic nuclear factor-kappa B (NF-κB) which is up-regulated along with elevated PICs and angiotensin II (ANG II) in the PVN of HF rats. These findings suggest that the NF-κB mediates the cross-talk between RAS and PICs in the PVN in HF. The further studies indicate that the interaction between AT_1_-R and NF-κB in the PVN contributes to oxidative stress and sympathoexcitation by modulating neurotransmitters in heart failure, and the superoxide activates NF-κB in the PVN and contributes to neurohumoral excitation. In conclusion, the neurohumoral excitation in HF is based on the interaction of RAS, PICs, ROS, NF-κB and neurotransmitters in the PVN; and the activated NF-κB in the PVN modulates the neurotransmitters and contributes to sympathoexcitation in rats with heart failure.

## Review

### Introduction

Heart failure (HF) secondary to left ventricular systolic dysfunction is characterized by low cardiac output and neurohumoral excitation (NHE). In the meantime, the sympathoexcitation is considered as an important characteristic of heart failure [1]. A growing number of experimental studies suggest that interventions at the central nervous system (CNS) level are beneficial during heart failure [2, 3]. Animals with HF have neurochemical abnormalities in the brain and profoundly affect sympathetic drive in heart failure. The increase in sympathetic nerve activity (SNA) in HF is due to an imbalance between inhibitory and excitatory mechanisms within specific areas in the CNS such as the paraventricular nucleus (PVN) of the hypothalamus [4]. Some studies show that the PVN is an important central site for integration of sympathetic nerve activity [5, 6] and is a significant area in cardiovascular control [7, 8]. The baseline sympathetic outflow mediated by the PVN mainly depends on the spontaneous activity of preautonomic neurons, and *via* the axons project to the intermediolateral cell column (IML).

Previous studies show that angiotensin II (ANG II), angiotensin II type 1 receptor (AT_1_-R), proinflammatory cytokines (PICs), NAD(P)H oxidase, and nuclear factor-kappa B (NF-κB) all appear to be potential targets for PVN interventions that might substantially reduce the adverse peripheral effects of sympathetic nerve activity and neurohumoral regulation in heart failure. So a comprehensive understanding of the PVN will enhance our ability to treat the HF condition and its cardiovascular complications, and help in reducing the deleterious effects of chronic sympathoexcitation in this disease state.

Based on evidence from other laboratories and our laboratory, this review highlights some important mechanisms in the PVN that may contribute to the exaggerated sympathetic outflow and the neurohumoral excitation commonly observed in HF.

### Renin-angiotensin system in heart failure

Heart failure is a consummate example of multisystem disorder and is characterized by neurohumoral excitation. The consequences of NHE include increased sympathetic activity, enhanced renal sodium and water reabsorption, and decreased renal perfusion resulting in the activation of a number of peptides including those components of the renin-angiotensin system (RAS). The RAS is activated in HF and leads to vasoconstriction, volume accumulation and exaggerated sympathetic nerve activity. The RAS has been implicated in the central processing of sympathetic nerve activity [9–12]. Recent studies demonstrated that RAS is activated in the PVN of heart failure rats and increased ANG II causes PVN neuronal activation [13–16]. ANG II had been shown as a significant factor in the central regulation of arterial blood pressure (BP), which could bind to AT_1_-R cross the weak or absent blood–brain barrier and subsequently activate the brain RAS. The permissive role of ANG II on sympathoadrenal activation is mediated by the same AT_1_-R which mediates the central effects of ANG II. Because the AT_1_-R is thought to be the primary receptor involved in most of the biological effects of ANG II, the degree and mechanism by which is expressed may provide important insight into the sympathoexcitatory process and the development of novel centrally acting therapeutic agents. Previous studies have demonstrated that AT_1_-R is present in the PVN [15, 17] and mRNA levels for the AT_1_ receptor are high in the PVN. Blockade of RAS components modulates PVN neurotransmitters and decreases sympathetic activity, indicating a role for the central nervous system RAS in sympathoexcitation in HF [13, 18]. Findings from our laboratory and others indicate that cytokines interact with RAS both in the central and peripheral nervous systems [13, 19–21]. Brain AT_1_-R may influence sympathetic activity by regulating the release of pro-inflammatory cytokines into the circulation. Cytokine blockade decreases circulating ANG II levels, conversely, RAS blockade attenuates circulating cytokine levels [22–24]. Therefore, it is plausible to suggest that an interaction between cytokine and AT_1_-R within the PVN might modulate neurotransmitters and contribute to sympathoexcitation in HF.

### Proinflammatory cytokines in the PVN in heart failure

A growing body of evidence suggests that heart failure is related with neurohumoral excitation as well as immune abnormal. The products of immune activation are the proinflammatory cytokines (PICs). These PICs include tumor necrosis factor-alpha (TNF-α), interleukin-1β (IL-1β) and IL-6, which are released into the circulation early after myocardial infarction (MI). Although several PICs are up-regulated in HF, we have focused on TNF-α, which appears early in the cytokine cascade [25], since it is generally the first cytokine that is up-regulated in diseases, and it also induces the production of several other cytokines. Findings from Felder’s laboratory suggest that TNF-α increases in the blood, brain and heart within minutes after acute MI and continues to rise over the ensuing weeks, and IL-1β has a similar pattern of early appearance after MI [26]. Chronic treatment with pentoxifylline (anti-inflammatory/anti-cytokine) inhibits PICs synthesis [27–30] and prevents the increases in TNF-α in brain, heart and plasma measured 4 weeks after MI [26]. The PVN is particularly sensitive to the influences of inflammatory stress. Injection of TNF-α into the PVN increased sympathetic activity, suggesting a direct role of TNF-α in sympathetic activity. Systemic TNF-α increases the activity of PVN neurons and contributes to increased sympathetic activity, chronic infusion of PICs synthesis blocker decreased sympathetic activity in HF rats [26, 31, 32]. There are two possible mechanisms for TNF-α increased expression in the PVN: (1) circulating TNF-α into the brain through the circumventricular organs (CVOs) which are specialized brain regions that lack a blood–brain barrier; (2) activated microglia in the brain can synthesize TNF-α. Blood-borne cytokines act upon receptors in the microvasculature of the brain to induce COX-2 activity and the production of prostaglandin E_2_, which penetrates the blood–brain barrier to activate the sympathetic nervous system [33]. Increased TNF-α in autonomic regulatory regions of the brain alters the production of superoxide and nitric oxide, contributing to fluid imbalance and sympathoexcitation in heart failure [34]. This is also supported by our findings that PVN infusion of a TNF-α blocker pentoxifylline (PTX) or etanercept (ETN) attenuates the increase in renal sympathetic nerve activity (RSNA), decreases AT_1_-R expression and modulates neurotransmitters thereby attenuating sympathoexcitation in HF rats [35].

### Neurotransmitters in the PVN in heart failure

A number of excitatory and inhibitory neurotransmitters converge in the PVN to influence its neuronal activity [5]. Among these neurotransmitters are glutamate, norepinephrine (NE), and gamma-amino butyric acid (GABA). Glutamate is a well-known excitatory neurotransmitter in the CNS. It has been reported that functional glutamate receptors are expressed in the PVN [36–39] and are involved in cardiovascular reflexes [40, 41]. It has been shown that sympathetic hyperactivity in HF rats is associated with increased extracellular NE in the PVN [42, 43]. NE plays a critical role in the pathophysiology of HF [44, 45]. GABA is a well-known inhibitory neurotransmitter in the CNS. A large body of evidence suggests that GABA plays an important role in central sympathetic and cardiovascular regulation [46, 47], which is a dominant inhibitory neurotransmitter within the PVN. Considerable evidence suggests that the PVN is one of the sites in which the cardiovascular effects of GABA are elicited. Studies from Patel’s laboratory demonstrate that inhibitory mechanisms of sympathetic regulation within the PVN *via* GABA were reduced in HF rats [48]. The alterations seen in HF may induce an imbalance between the inhibitory and excitatory neurotransmitters in the PVN and influence sympathetic outflow [49, 50]*.* PICs were increased in the PVN of HF rats and increased PICs in the PVN cause an imbalance in PVN neurotransmitters and contribute to sympathoexcitation in heart failure [21, 35]. Rats with HF or sham-operated control (SHAM) rats were treated for 4 weeks with a continuous intracerebroventricular (ICV) infusion of the TNF-α blockers PTX, ETN or vehicle. HF rats had increased neuronal excitation accompanied by higher levels of glutamate, NE, and tyrosine hydroxylase (TH), and lower levels of GABA and 67-kDa isoform of glutamate decarboxylase (GAD67) in the PVN when compared with SHAM rats. Renal sympathetic nerve activity (RSNA) was also increased in HF rats. After the ICV treatment with low doses of PTX or ETN attenuated, and high doses prevented, increases in levels of glutamate, NE, and TH, and decreases in levels of GABA and GAD67 in the PVN of HF rats. These studies from our laboratory clearly indicate that the effects of PICs on the exaggerated sympathetic activity in HF *via* modulating neurotransmitters in the PVN.

### Reactive oxygen species in the PVN in heart failure

Although there are differences in sympathetic outflow to various vascular beds in the HF state, it is generally well accepted that sympathoexcitation is a global phenomenon [51]. Furthermore, HF has been viewed as a proinflammatory state as well as a condition characterized by high levels of oxidative stress [52–54]. In patients and animals with HF, increased oxidative stress has been shown to occur in many tissues, including the heart and brain [55–59]. The NAD(P)H oxidase is a multi-subunit enzyme that catalyzes the reduction of molecular oxygen to form superoxide O_2_^•^ˉ. NAD(P)H oxidase function appears to be required for processes such as neuronal signaling and central cardiovascular homeostasis, but overproduction of reactive oxygen species (ROS) contributes to neurodegeneration and cardiovascular diseases [60]. TNF-α can induce activation of NAD(P)H oxidase leading to enhance oxidative stress [61] and mediate NAD(P)H oxidase-derived superoxide production during heart failure [60]. Furthermore, overproduction of ROS within brain cardiovascular regions such as the PVN induced the overexpression of several NAD(P)H oxidase subunits including NOX-2 and NOX-4 [62]. Under pathological conditions, the excess O_2_^•^ˉ in the brain made the disequilibrium of oxidation and antioxidation resulting in augmented the sympathoexcitaion in heart failure.

However, studies from our laboratory indicate that the treatment with tempol (a superoxide scavenger) not only decreased sympathetic activity, but also eliminated the redundant O_2_^•^ˉ, restored the balance between oxidation and antioxidation, decreased the expression of RAS component AT_1_-R, and ameliorated heart failure [20].

### Nuclear factor-kappa B in the PVN in heart failure

The neurohumoral mechanisms and immune-mediated mechanisms have been shown to play important roles in the pathophysiology of HF, and excess PICs [14] and RAS [63] are both present in the cerebrum cardiovascular region and contribute to neurohumoral excitation in HF.

Nuclear factor-kappa B (NF-κB) is present in central nervous system (CNS) neurons and plays an important role in inflammatory response, and is a potent inducer of PICs and oxidative stress contributes to the pathophysiology of multiple disease states [64]. The activated NF-κB pathway has been shown to be the major regulator facilitating the synthesis of several different injury-responsive cytokines in neurons, such as TNF-α, IL1-β, IL-6 [65–67]. AT_1_-R is the important product mediated by cytoplasmic NF-κB [67]. Translocation of activated NF-κB to nuclei regulates the synthesis of AT_1_-R in neurons [68]. Furthermore, NF-κB can mediate the cross-talk between RAS and PICs in the PVN in HF [20]. In the PVN, the activated NF-κB also contributes to NAD(P)H oxidase-dependent oxidative stress and sympathoexcitation in HF rats [69, 70]. In return, NF-κB is up-regulated along with PICs activation in the PVN of ischemia-induced heart failure rats [71]. RAS and superoxide activate NF-κB in the PVN and contribute to neurohumoral excitation [71–73]. In addition, oxidative stress mediates the up-regulation of brain renin-angiotensin system (RAS) in ischemia-induced heart failure [71].

**Figure 1:**
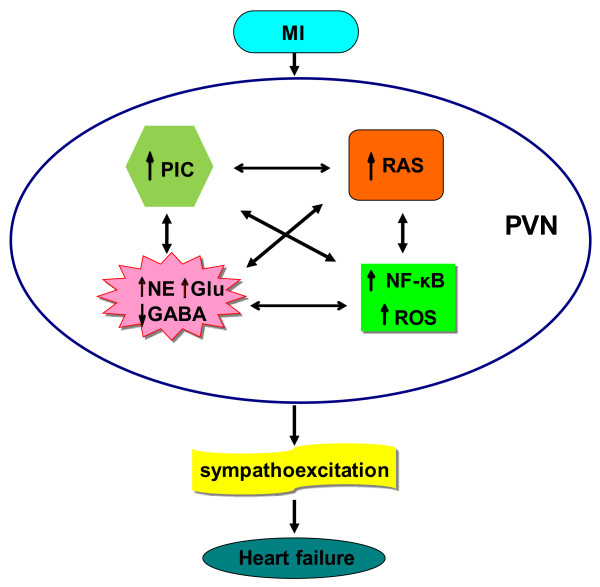
**The interaction among RAS, PICs, ROS, NF-κB and neurotransmitters in the PVN contributes to sympathoexcitation in heart failure.**

Previous work demonstrated a GABA-mediated inhibitory mechanism in the PVN contributing to sympathoexcitation in HF rats [70]. Moreover, in HF, endogenous GABA-mediated inhibition is decreased, due to the increased neuronal activity of the PVN. PVN infusion of AT_1_-R blocker losartan or pyrrolidine dithiocarbamate (PDTC, a specific NF-κB inhibitor) attenuated the decreases in GABA, and the increases in gp91^phox^ (a subunit of NAD(P)H oxidase), NF-κB activity, glutamate and NE, in the PVN of HF rats, and also attenuated the increases in RSNA and plasma PICs and NE. Based on these studies, it is clear that interaction between AT_1_-R and NF-κB in the PVN contributes to oxidative stress and sympathoexcitation by modulating imbalance between excitatory and inhibitory neurotransmitters in the PVN of HF rats [74]. PVN infusion of SN50 (a competitive inhibitor of the translocation of NF-κB to the nucleus) prevented the decreases in PVN GABA and GAD67, and the increases in RSNA and PVN glutamate, NE, TH, superoxide, gp91^phox^, phosphorylated IKKβ and NF-κB p65 activity observed in heart failure rats. These findings suggest that the activated NF-κB contributes to sympathoexcitation in rats with ischemia-induced heart failure [63]. Figure 1 provides a schematic overview and summarizes the relationship of PICs, ROS, RAS and NF-κB in the PVN, which modulate sympathetic activity in heart failure.

## Conclusion

Neurohumoral excitation has been recognized as one of the hallmarks during the development of heart failure (HF), a large number of factors such as ANG II, PICs, ROS and NF-κB signaling are activated and cause an imbalance in PVN neurotransmitters and contribute to sympathoexcitation in heart failure. The precise mechanisms by which these factors become sensitized in HF are needed to be explored. Several possibilities, including indirect effects mediated by PICs, NAD(P)H oxidase-dependent generation of superoxide and/or up-regulation of the brain RAS, and possibly even direct effects mediated by NF-κB signalling pathways. A clear indication of this phenomenon is illustrated in Figure 1. In the PVN, NF-κB activation seems to play an important role in sympathoexcitation in HF. Interaction among PICs, ROS, RAS and NF-κB in the PVN modulates sympathetic nerve activity. Targeting NF-κB modulation and its downstream regulatory molecules in the PVN provides a roadmap for tackling possible therapeutic strategies to the improvement of central neural control of sympathetic nerve activity in HF. We conclude that increased brain PICs in HF, either directly, or *via* an interaction with RAS, ROS and NF-κB, cause an imbalance between excitatory and inhibitory neurotransmitters in the PVN, thereby contributing to sympathoexcitation.

The review provided here clearly indicate the great potential for development of future therapies involving targets within the RAS, ROS, PICs, neurotransmitter and NF-κB. Further studies are needed to elucidate the significance of these mechanisms that may contribute but are not yet appreciated. And the functional neurons for the sympathoexcitation in the PVN in heart failure, such as the microglia, are needed to be investigated in the further studies.
